# Structure-Guided Engineering of a Complement Component C3-Binding Nanobody Improves Specificity and Adds Cofactor Activity

**DOI:** 10.3389/fimmu.2022.872536

**Published:** 2022-07-22

**Authors:** Henrik Pedersen, Rasmus Kjeldsen Jensen, Annette Gudmann Hansen, Steen Vang Petersen, Steffen Thiel, Nick Stub Laursen, Gregers Rom Andersen

**Affiliations:** ^1^ Department of Molecular Biology and Genetics, Aarhus University, Aarhus, Denmark; ^2^ Department of Biomedicine, Aarhus University, Aarhus, Denmark

**Keywords:** single-domain antibody, complement system, alternative pathway, inhibitor, Factor H

## Abstract

The complement system is a part of the innate immune system, where it labels intruding pathogens as well as dying host cells for clearance. If complement regulation is compromised, the system may contribute to pathogenesis. The proteolytic fragment C3b of complement component C3, is the pivot point of the complement system and provides a scaffold for the assembly of the alternative pathway C3 convertase that greatly amplifies the initial complement activation. This makes C3b an attractive therapeutic target. We previously described a nanobody, hC3Nb1 binding to C3 and its degradation products. Here we show, that extending the N-terminus of hC3Nb1 by a Glu-Trp-Glu motif renders the resulting EWE-hC3Nb1 (EWE) nanobody specific for C3 degradation products. By fusing EWE to N-terminal CCP domains from complement Factor H (FH), we generated the fusion proteins EWEnH and EWEµH. In contrast to EWE, these fusion proteins supported Factor I (FI)-mediated cleavage of human and rat C3b. The EWE, EWEµH, and EWEnH proteins bound C3b and iC3b with low nanomolar dissociation constants and exerted strong inhibition of alternative pathway-mediated deposition of complement. Interestingly, EWEnH remained soluble above 20 mg/mL. Combined with the observed reactivity with both human and rat C3b as well as the ability to support FI-mediated cleavage of C3b, this features EWEnH as a promising candidate for *in vivo* studies in rodent models of complement driven pathogenesis.

## Introduction

The complement system comprises a proteolytic cascade that can be activated *via* the lectin pathway (LP), the classical pathway (CP), or the alternative pathway (AP). The three pathways converge at the cleavage of complement component C3, which results in covalent deposition of the cleavage product C3b on the activating surface. Activator-bound C3b associates with the zymogen Factor B (FB), which is subsequently cleaved by the protease Factor D (FD). This establishes the AP C3 convertase (1) that cleaves C3 to C3b, whereby the AP amplifies the outcome of the LP and CP (2, 3). The internal thioester bond of C3 undergoes spontaneous hydrolysis, forming C3(H_2_O) that functionally resembles C3b and allows assembly of a fluid-phase C3 convertase (4). If the complement cascade proceeds unhindered through the terminal pathway, the cascade may lead to the assembly of the membrane attack complex that facilitates cell lysis (5).

To prevent complement-induced damage of host cells, regulators control the accumulation of C3b (6). One of these, FH, is a fluid phase regulator that holds two functions: i) it accelerates the decay (dissociation) of the AP C3 convertase, and ii) it serves as cofactor for factor I (FI), which degrades C3b to iC3b. FH comprises 20 complement control protein (CCP) domains, where the four N-terminal domains (CCP1-4) harbor both functions of FH (7, 8). Mutations in the human FH that compromises function may predispose for complement-related diseases (9), underscoring the importance of FH as a central regulator of complement activation. Hence, replacing patients’ compromised FH with recombinant FH would sustain a putative therapeutic strategy. However, FH replacement therapy remains unfeasible, because of difficulties in large scale recombinant expression of full-length FH (10–12). As an alternative, minimized versions of FH (mini-FH), that comprise the CCP1-4 and CCP19-20 domains (13, 14) have been investigated. Similarly, Nichols *et al.* described the Newcastle mini-FH, which comprises the CCP1-5 and CCP18-20 domains (15). These mini-FH proteins accelerate the decay of the AP C3 convertase and serve as cofactor for the FI-mediated cleavage of C3b (13–15) and constitute interesting new modalities for complement inhibition.

**Figure 1:**
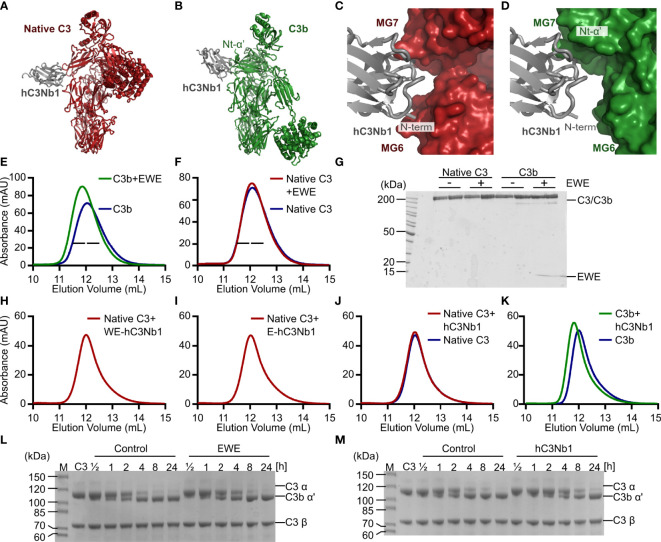
Design and validation of EWE. **(A, B)** Structural comparison of the hC3Nb1 (grey) in complex with native C3 (red) and C3b (green). C3 and C3b are aligned on their MG-ring. **(C, D)** Comparison of the binding interface of hC3Nb1 in complex with C3 and C3b. The structures in panels **(C, D)** are aligned on the hC3Nb1 molecule. PDB entries are 6RU5 for the C3:hC3Nb1 complex and 6EHG for the C3b:hC3Nb1 complex. **(E, F)** SEC-based analyses of the interaction of EWE with C3b and native C3. **(G)** SDS-PAGE analysis of peak fractions from panels **(E, F)**, indicated by bars. **(H–J)** SEC-based analyses of the complex-formation between native C3 and WE-hC3Nb1, E-hC3Nb1, and hC3Nb1. **(K)** SEC-based analysis of the interaction between hC3Nb1 and C3b. **(L, M)** Cleavage of C3 by CVFBb was monitored in presence or absence of EWE or hC3Nb1. *mAU*, milli absorbance units. *M*, molecular weight marker.

We previously showed that the nanobody hC3Nb1 is a strong inhibitor of the AP (16). However, at least two caveats complicate the therapeutic use of hC3Nb1: firstly, hC3Nb1 was found to interact with native C3 present at a concentration of ~7 μM in plasma (17, 18); secondly, hC3Nb1 inhibits FH-mediated cleavage of C3b by FI, which could lead to a buildup of C3b on complement activators. In native C3, the epitope of hC3Nb1 is located on the macroglobulin (MG)6-MG7 domains of C3 (19). Upon cleavage of C3 structural rearrangements separate the two MG domains (**Figures 1A–D**), and the nanobody no longer interacts with the MG6 domain (16). Interactions with the α’ chain (Nt-α’) of C3b exposed upon C3 cleavage possibly compensate for the lost interaction with MG6 (16). Interestingly, the altered binding site of hC3Nb1 exposes its N-terminus, which interacts with the MG6 domain when the nanobody is in complex with C3 (19). This binding site of hC3Nb1 inspired us to develop modified nanobodies that does not bind native C3 to allow the nanobody to specifically target sites of complement activation. In this study, we expand our toolbox of complement targeting nanobodies (reviewed in (20)) with the development of a new version of hC3Nb1, called EWE (due to the N-terminal addition of the sequence Glu-Trp-Glu), that specifically binds C3 degradation fragments and inhibits the AP. We present the fusion proteins EWEnH and EWEµH, which comprise EWE fused to either CCP2-4 or CCP2-4 and CCP19-20 of FH, respectively, and restore cofactor activity. These fusion proteins provide new modalities for efficient inhibition of the AP, while sustaining the degradation of C3b.

## Materials and Methods

### Protein Purification

A Glu-Trp-Glu -motif was introduced N-terminal to the hC3Nb1 sequence in the pET-22b(+) plasmid encoding the nanobody. The resulting EWE nanobody was expressed in LOBSTR (21) cells. All nanobodies were purified as previously described (16). In brief, LOBSTR cells harboring the nanobody-encoding plasmid were grown to an A_600nm_ of 0.6, nanobody expression was induced by 1 mM Isopropyl β-D-1-thiogalactopyranoside, and the cells were grown for 16 h at 18°C. Next, cells were pelleted by centrifugation, resuspended in binding buffer (20 mM Tris pH 8.5, 500 mM NaCl, 20 mM imidazole pH 8, 0.5 mM EDTA), lysed by sonication, and the cell debris was removed by centrifugation. The cleared lysate was loaded onto a HisTrap FF Crude column (GE Healthcare), the column was washed in binding buffer, and the nanobody was eluted by binding buffer supplemented with 400 mM imidazole pH 8. The nanobody was dialyzed against 20 mM Na Acetate pH 5.5, 50 mM NaCl for 16 h at 4°C and subsequently loaded onto a 1 ml Source 15S column (GE Healthcare). The nanobody was eluted by a linear gradient from 50-350 mM NaCl over 35 column volumes and further purified by size exclusion chromatography (SEC) on a Superdex 75 column (GE Healthcare) equilibrated in 20 mM HEPES pH 7.5, 150 mM NaCl. An avi-tagged version of EWE was purified similarly and biotinylated as described (22).

For expression of EWE and hC3Nb1 in mammalian cell, the hC3Nb1 was cloned into a pcDNA 3.1 vector, and the ‘EWE’ motif was subsequently introduced. The resulting constructs were transiently expressed in HEK293 cells using polyethylenimine transfection. Upon 5 days of incubation, the conditioned media was harvested, added Tris pH 8.5 to a final concentration of 50 mM, and loaded onto a HisTrap excel column (GE Healthcare) equilibrated in 20 mM Tris pH 8.5, 500 mM NaCl. The protein was eluted in 20 mM Tris pH 8.5, 500 mM NaCl, 400 mM imidazole pH 8, and dialyzed against 20 mM Na Acetate pH 5.5, 50 mM NaCl for 16 h at 4°C. The dialyzed proteins were subjected to ion exchange and SEC as described above for bacterially expressed nanobodies.

The EWEµH construct in pcDNA3.1 was generated by Genscript (**Supplementary Table 1**). The EWEnH encoding plasmid was generated from EWEµH by inserting a 6xHis-tag and a stop-codon before the linker joining CCP4 and CCP19 (**Supplementary Table 1**). EWEnH and EWEµH were transiently expressed in HEK293 cells using polyethylenimine transfection. Upon five days of incubation, the conditioned media was harvested and loaded onto a HisTrap excel column equilibrated in 20 mM Tris pH 8.5, 500 mM NaCl. The protein was eluted by application of 20 mM Tris pH 8.5, 500 mM NaCl, 400 mM imidazole pH 8. The eluted protein was dialyzed against 20 mM Tris pH 8.5, 50 mM NaCl for 16 h at 4°C, then applied to a 9 mL Source 15Q (GE Healthcare) equilibrated in 20 mM Tris pH 8.5, 20 mM NaCl. The protein was eluted by a linear gradient from 50-500 mM NaCl over 60 mL. Next, the protein was purified by SEC on a Superdex 200 increase column (GE Healthcare) equilibrated in 20 mM HEPES pH 7.5, 150 mM NaCl. Mini-FH was prepared from transiently transfected HEK293 cells as described (19).

Native C3 was purified from plasma as described (16). C3 methylamine (C3MA) was generated from freshly purified native C3 by treatment with 100 mM methylamine at pH 8.5, and C3b was generated from native C3 by trypsin cleavage as described (22), whereas iC3b was generated as described in (23). Rat C3 was purified from rat EDTA plasma generously provided by Emma Faddy (Department of Infectious Diseases, Aarhus University). Rat plasma was diluted 1:1 to obtain a final concentration of 20 mM Tris pH 7.5, 20 mM NaCl, 5 mM EDTA, 1 mM benzamidine, 0.8 µg/mL pancreatic trypsin inhibitor, and 2.5 mM Pefabloc. The plasma was added 0.041 volume of 50% (w/v) PEG6000, stirred gently for 20 min, then centrifuged at 3500×g for 20 min. The supernatant was subjected to a second PEG precipitation step by addition of 0.11 volume of 50% PEG6000. The resulting pellet was resuspended in C3 purification buffer (20 mM Tris pH 7.5, 20 mM NaCl, 5 mM EDTA, 1 mM benzamidine, 0.8 µg/mL pancreatic trypsin inhibitor, 2.5 mM Pefabloc), then loaded onto a HiTrap Q FF column (GE Healthcare) equilibrated in 20 mM Tris pH 7.5, 20 mM NaCl, 5 mM EDTA. The protein was eluted by a linear gradient from 20-300 mM NaCl. The protein was dialyzed against 20 mM HEPES pH 7.5, 50 mM NaCl at 4°C, then loaded onto a 9 mL Source 15Q column. The protein was eluted by a linear gradient from 0-500 mM NaCl over 60 mL. The protein was diluted 1:4 with 20 mM MES pH 6.0 and applied to a 1 mL Mono S column (GE Healthcare) equilibrated in 20 mM MES pH 6.0, 30 mM NaCl. The protein was eluted in a gradient from 30 to 500 mM NaCl over 40 mL. Finally, the C3 was purified by SEC on a 24 ml Superdex 200 increase column (GE Healthcare) equilibrated in 20 mM HEPES pH 7.5, 150 mM NaCl. To generate rat C3b, rat C3 was cleaved by cobra venom factor (CVF)Bb in 20 mM HEPES pH 7.5, 150 mM NaCl, 2 mM MgCl_2_. The CVFB was prepared by mixing recombinant FB, carrying the D279G mutation that stabilizes the convertase (24), with a 1.2-fold molar excess of CVF. Subsequently, 10% (w/w) FD (Complement Technology) was added to the proconvertase and the reaction was incubated for 15 minutes at room temperature, then for 10 min on ice. The resulting CVFBb was added a 50-fold molar excess of rat C3 and incubated for 30 min at 37°C. The resulting rat C3b was used immediately upon cleavage. CVF was prepared as described (25), whereas recombinant human FB D279G, was prepared according to (26). FD was purified as described (22) or purchased at Complement Technology, where indicated.

### Analytical Size Exclusion Chromatography

For analytical SEC assays, purified C3 or C3b was mixed with a two-fold molar excess of EWE or hC3bNb1. The samples were diluted to 400 µL in 20 mM HEPES pH 7.5, 150 mM NaCl and incubated for 5 min on ice. The samples were loaded onto a 24 ml Superdex 200 increase column (GE Healthcare) equilibrated in 20 mM HEPES pH 7.5, 150 mM NaCl.

### C3 Cleavage Assay

The C3 cleavage assay was conducted in 20 mM HEPES pH 7.5, 150 mM NaCl, 2 mM MgCl_2_. The CVFBb proconvertase was prepared as described above. Meanwhile, native C3 was mixed with a two-fold molar excess of EWE or hC3Nb1. A 10-fold molar excess of native C3 to FB was added to the CVFBb mixture, and the reactions were incubated on ice for 30 min, 1 h, 2 h, 4 h, 8 h, or 24 h.

### Crystallization of the EWE:C3b Complex

C3b was concentrated to 6.5 mg/mL and mixed with a two-fold molar excess of EWE. Drops were formed by mixing 200 nL of the complex in 20 mM HEPES pH 7.5, 150 mM NaCl with 200 nL of the reservoir condition containing 112 mM Sodium Citrate pH 8, 28 mM Sodium Citrate pH 6.0, 12.25% (w/v) PEG 2000 MME, 70 mM Imidazole pH 7, 60 mM Ammonium acetate, 70 mM TRIS pH 8.5, and 13.5% (v/v) 2-Methyl-2,4-pentanediol. Crystals were grown by sitting drop vapor diffusion at 19°C. Crystals were cryo protected in the reservoir condition supplemented with 5% (v/v) glycerol and data collected at the P13 beamline at the Petra III synchrotron. Data was processed with XDS (27) and phased by molecular replacement using the C3b:hC3Nb1 (PDB entry 6EHG) structure (16) in Phaser (28). The model was iteratively manually rebuilt in Coot (29) and refined using Phenix.refine (30). Figures were prepared in PyMol (version 2.3.0).

### Surface Plasmon Resonance

All experiments were performed on a BIAcore T200 instrument (GE Healthcare) in surface plasmon resonance running buffer (20 mM HEPES pH 7.5, 150 mM NaCl, 0.05% Tween 20) at a flow rate of 30 µL/min at 25°C. Streptavidin (5 µg/mL) diluted in Na acetate pH 4.5 was immobilized on a CM500M chip (XanTec Bioanalytics GmbH) to approximately 300 response units (RU) using an amine coupling kit (GE Healthcare). A solution of 30 µg/mL biotinylated C-terminally avi-tagged nanobody in running buffer was bound to the streptavidin surface. Native C3, C3b and C3MA were subsequently injected in concentrations ranging from 0.4 nM to 200 nM. The nanobody surface was regenerated by 100 mM glycine pH 2.7 for three cycles of 10 seconds. Signal from reference surface and the average of the 0 nM analyte sensorgrams was subtracted prior to data-fitting. Due to spikes in sensorgrams, signals from 3 seconds in the start of the association phase and 2 seconds in transition from the association to dissociation were excluded. The data were fitted using the non-linear fitting tool, ‘Association then dissociation’ with shared k_on_ and k_off_ for all datasets as constrains in the GraphPad Prism (version 6.01) software. Values are presented as mean ± S.E. from three independent experiments.

### Bio-Layer Interferometry

All experiments were performed in bio-layer interferometry (BLI) running buffer (20 mM HEPES pH 7.5, 150 mM NaCl) using an Octet RED96 instrument (FORTÉBIO Pall Corporation) at 30°C and 1000 rpm of shaking. For the binding kinetic experiments, EWE, EWEµH or EWEnH at 10 µg/mL was immobilized on anti-penta his (His1K) biosensors (Sartorius). EWEnH was transferred into 0, 6.25, 12.5, 25, 50, and 100 nM iC3b or 0, 3.125, 6.25, 12.5, 25, 50, 100 nM C3b. EWEµH was transferred into 0, 3.125, 6.25, 12.5, 25, 50 nM iC3b or 0, 1.5625, 3.125, 6.25, 12.5, 25, 50 nM C3b. EWE was transferred into 0, 3.125, 6.25, 12.5, 25, 50 nM iC3b or 0, 1.5625, 3.125, 6.25, 12.5, 25, 50 nM C3b. The association period was 180 seconds, followed by a 180 second dissociation step in the BLI running buffer. Sensors were regenerated using five washes in 20 mM Na acetate pH 5.5, 1 M NaCl for 10 seconds, and BLI running buffer for 10 seconds. The sensorgrams were normalized by subtracting the 0 nM analyte sensorgram from each dilution series. The normalized sensorgrams were fitted to a 1:1 binding mode in the GraphPad Prism (version 6.01) software. Values are presented as mean ± S.E. from the indicated number of independent experiments. To analyze the interaction between hC3Nb1 variants and C3 or C3b, nanobody at 10 µg/mL was immobilized on anti-penta his (His1K) biosensors (Sartorius). The sensors were transferred into 50 nM C3 or C3b for 180 seconds followed by 180 seconds dissociation period. The sensorgrams were normalized by subtracting 0 nM analyte sensorgrams.

### C3b Cleavage Assays

FI mediated degradation assays were performed in 20 mM HEPES pH 7.5, 150 mM NaCl. For EWE and hC3Nb1, C3b was incubated with a 1.2-fold molar excess of nanobody for 5 min. Next, 1:100 (w/w) FI (Complement Technology) and 1:500 (w/w) FH (Complement Technology) relative to C3b were added to the C3b-nanobody samples, and the samples were incubated for the indicated time at 37°C. In the initial EWEµH cleavage assay, C3b was mixed with 0.5% (w/w) FI (Complement Technology) and a two-fold molar excess (to C3b) of mini-FH, EWEµH, or both mini-FH and EWE. In the assay comparing the fusion proteins, C3b was mixed with EWEµH or EWEnH in a molar ratio of 1:0.5 or 1:2. The samples were incubated for 5 min at 4°C, then 1% (w/w) FI (Complement Technology) relative to C3b was added. The samples were incubated at 37°C for the indicated time. Cleavage assay at 1:0.5 ratio was performed twice, and the 1:2 ratio cleavage assay was performed three times with similar results. The assay assessing the FI cleavage of rat C3b in the presence of EWEµH or EWEnH was performed using a 1:2 molar ratio and was performed as described above. This experiment was performed twice.

### C3 Fragment Deposition Assays

Activation of the AP was followed by measuring the deposition of C3 fragments onto microtiter well surfaces. A 96-well plate was coated with 20 µg/mL zymosan in 50 mM sodium carbonate pH 9.6 and incubated overnight at 4°C. To block remaining protein binding sites, the plate was subsequently incubated in TBS/Tween (10 mM TRIS pH 7.4, 145 mM NaCl, 0.05% Tween 20) with 1 mg/mL human serum albumin for 1 h at room temperature. The plate was washed three times in TBS/Tween and added 100 µL normal human serum diluted to 11% (v/v) in AP deposition buffer (10 mM HEPES pH 7.5, 140 mM NaCl, 10 mM EGTA, 5 mM MgCl_2_) either in the presence or absence of nanobody in concentrations from 4.9-1200 nM. The plate was incubated for 1 h at 37°C in a humid chamber and subsequently washed three times in TBS/Tween. Next, 100 µL 0.75 µg/mL biotinylated anti hC3d (DAKO) in TBS/Tween was added to the wells followed by a 16 h incubation period at room temperature. The wells were washed three times in TBS/Tween, then incubated with 1 µg/mL streptavidin-Eu (PerkinElmer) for 1 h at room temperature. Subsequently, the plate was washed three times in TBS/Tween, followed by incubation with enhancement buffer (Ampliqon) for 2 min. The signal of the europium in the wells was subsequently read by time-resolved fluorometry on a VICTOR 5 plate reader (PerkinElmer) and was given as counts per second.

### AP Hemolysis Assay

To analyze the effect of the nanobody constructs on AP mediated hemolysis, 11% normal human serum was mixed with 1.26, 0.42, 0.4, 0.05, 0.02 and 0.005 µM of the indicated nanobodies. All dilutions were made in hemolysis buffer (20 mM HEPES pH 7.4, 150 mM NaCl, 5 mM MgCl_2_, 10 mM EGTA with 0.1% gelatin). A positive control without nanobody was included as well as a negative control without serum. A 20 µL volume of the nanobody-serum mixture and 10 µL 6% v/v rabbit erythrocytes in hemolysis buffer was added to a 96 V-bottom plate (Thermo 249662). The plate was incubated at 37°C for 2 h, and then complement activity was stopped by the addition of 60 µL cold 154 mM NaCl, 5 mM EDTA. The non-lysed erythrocytes were pelleted by centrifugation at 200×g for 20 min and 70 µL of the supernatant was transferred to a 96-well plate. The absorbance was measured at 405 nm on a VICTOR 3 Multilabel Plate Counter (PerkinElmer).

### Mass Spectrometry

Purified protein was diluted in 0.1% TFA and mixed 1:1 (vol:vol) with 2,5-dihydroxyacetophenone (0.1 M in 20 mM ammonium dihydrogen citrate and 75% (v/v) EtOH) (31). The material was spotted onto a stainless steel target plate and allowed to dry. The spectra were recorded in positive and linear mode using an AutoFlex Smartbeam III instrument (Bruker) calibrated by external calibration (Peptide calibration standard I, Bruker Daltronics).

### Ultrafiltration Test

EWEnH and EWEµH was concentrated in a Vivaspin 500 centrifugal concentrator (Sartorius) at 4°C. EWEnH was diluted 10-fold in 20 mM HEPES pH 7.5, 150 mM NaCl and the A_280nm_ was measured on a Nanodrop ND-10000 spectrophotometer (Saveen Werner), whereas EWEµH was not diluted prior to determination of the concentration. Next, the EWEnH was loaded onto a Superdex 200 increase column (GE Healthcare) equilibrated in 20 mM HEPES pH 7.5, 150 mM NaCl to analyze for aggregation.

## Results

### Structural Considerations for the Design of EWE

We previously described the C3-specific nanobody hC3Nb1 (16) and also the crystal structures of hC3Nb1 in complex with native C3 (**Figure 1A**) (19) and C3b (**Figure 1B**) (16). In complex with native C3, the complementarity-determining region (CDR)2 and CDR3 of the nanobody interacts with MG7 of C3, whereas the N-terminus of hC3Nb1 interacts with MG6 (**Figures 1A, C**) (19). In complex with C3b, hC3Nb1 similarly binds the MG7 domain, but also the newly exposed Nt-α’ (**Figures 1B, D**) (16). In contrast to the complex formed with C3, the N-terminus of hC3Nb1 is solvent accessible when bound to C3b (**Figure 1D**). This structural difference led us to hypothesize that extension of the N-terminus might obstruct the binding of hC3Nb1 to native C3, without compromising C3b binding. Hence, we added a Glu-Trp-Glu motif in the N-terminus of hC3Nb1 since this region protrudes toward negatively charged side chains in native C3. This modified hC3Nb1 nanobody is referred to as EWE.

To assess whether the N-terminal extension affects the ability of EWE to bind C3b, we used SEC. When mixing the nanobody with C3b, the chromatogram revealed that the presence of EWE conferred an earlier elution volume and a higher peak absorbance (**Figure 1E**), indicating complex formation. In contrast, we observed no difference in peak height or elution volume when EWE was incubated with C3 (**Figure 1F**), indicating that EWE does not bind native C3. SDS-PAGE analysis of the peak fractions from the two SEC analyses confirmed the formation of the EWE:C3b complex (**Figure 1G**).

To obtain insight into the conformation of the N-terminus of EWE, we co-crystallized EWE with C3b and collected X-ray diffraction data extending to 2.8 Å resolution (**Supplementary Table 2**). As expected, the overall structure of the EWE:C3b complex closely resembled the structure of the hC3Nb1:C3b complex (**Supplementary Figure 1A**). An inspection at the N-terminal region of EWE revealed only scattered electron density for the EWE-motif, indicating that the motif is flexible. The first residue that could be modelled was Gln4 (**Supplementary Figure 1B**), which corresponds to the N-terminal glutamine of hC3Nb1.

### The Parental hC3Nb1 may not Bind Native C3

We next sought to determine the minimal extension of hC3Nb1 that confers specificity towards C3 degradation products. We expressed and purified the two trimmed EWE variants WE-hC3Nb1 and E-hC3Nb1. In subsequent SEC analyses, we observed no change in the elution profile of C3 in the presence of either WE-hC3Nb1 or E-hC3Nb1 (**Figures 1H, I**), and no bands representing the nanobody were apparent by SDS-PAGE analysis of the peak fractions (**Supplementary Figure 2A**). In a control experiment, we analyzed the interaction between the parental nanobody hC3Nb1 and native C3. Surprisingly, we observed no interaction in SEC (**Supplementary Figures 1J**, **2A**), despite the clear shift in elution volume when mixing hC3Nb1 with C3b (**Figure 1K**), confirming the integrity of the hC3Nb1. These observations were surprising because of the high affinity, which we previously reported for the interaction between hC3Nb1 and native C3 (16).

To test whether the lack of C3-interaction of the hC3Nb1 was due to a modification of the nanobody, we analyzed the hC3Nb1 expressed in bacteria by mass spectrometry. The data indicate that the nanobody retains its N-terminal methionine (**Supplementary Figure 2B**) (*m/z* 14325.41 vs. *m/z*_calc_ 14327.9). This was also the case for the hC3Nb1 (W102A) mutant (*m/z* 14209.75 vs. *m/z*_calc_ 14212.8) (**Supplementary Figure 2C**) purified at the same time as the original hC3Nb1 (16). Unfortunately, the original preparation of hC3Nb1 was used up before this issue of altered C3 binding was identified. However, an inspection of the electron density for the hC3Nb1-C3 complex indicated that in the complex crystallized, hC3Nb1 did not retain this methionine (19). We hence purified the hC3Nb1 from mammalian cells using a construct that allows for the expression of hC3Nb1 without the N-terminal methionine. The subsequent mass spectrometry analysis indicated that, whereas the purified nanobody lacks the methionine, the mass was 17 Da lower than expected (**Supplementary Figure 2D**) (*m/z* 14093.02 vs. *m/z*_calc_ 14109.6), which we ascribed to formation of a pyroglutamate by the N-terminal glutamine residue. This modification could potentially affect the interaction with C3 and we hence purified two versions of hC3Nb1 incapable of forming the N-terminal pyroglutamate. In the first version, we mutated the N-terminal glutamine to asparagine (hC3Nb1 Q1N); in the second version, we deleted the N-terminal glutamine (hC3Nb1 ΔQ1). We expressed the two mutants in bacteria and analyzed the nanobodies by mass spectrometry, which confirmed the identity of the purified proteins (**Supplementary Figures 2E,F**) (Q1N *m/z* 14183.06 vs. *m/z*_calc_ 14182.7; ΔQ1 *m/z* 14071.11 vs. *m/z*_calc_ 14068.6). We then progressed to test the binding of the nanobodies to native C3 and C3b using BLI. We immobilized bacterially expressed hC3Nb1 or EWE and transferred the sensors into C3 or C3b, and the resulting sensorgrams revealed a large response when sensors were transferred into C3b, but not C3 (**Supplementary Figure 2G**). The same BLI experiment repeated with mammalian expressed hC3Nb1 and EWE gave a similar outcome (**Supplementary Figure 2H**). Lastly, we analyzed the hC3Nb1 ΔQ1 and Q1N mutants expressed in bacteria. Whereas both nanobodies conferred a strong signal when transferred into C3b, only a minuscule signal was observed when the nanobodies were transferred into native C3 (**Supplementary Figure 2I**). Overall, our BLI data indicate that none of our hC3Nb1 derivatives supported a strong interaction between hC3Nb1 and native C3. The lack of C3 binding by hC3Nb1 is unlikely to arise from glycan differences between native C3 preparations, because the binding site of hC3Nb1 lies far (> 40 Å) from the glycan sites on native C3 annotated in Uniprot CO3_HUMAN. Additionally, the lack of binding is unlikely to arise from aggregation as judged by SEC analysis or by incorrectly folded native C3, because our hC3Nb2 nanobody (22) bound both native C3 and C3b in all BLI experiments (not shown).

To analyze the specificity using an orthogonal approach, we tested the EWE and hC3Nb1 in a CVFBb cleavage assay since the parental hC3Nb1 delayed the cleavage of native C3 by CVFBb (16), indicating an interaction between hC3Nb1 and native C3. We incubated the CVFBb convertase with native C3 in the presence or absence of EWE or the parental hC3Nb1. The subsequent SDS-PAGE analyses indicated that the cleavage of C3 progressed with similar kinetics in the presence or absence of EWE (**Figure 1L**) and that hC3Nb1 only conferred a small delay of the cleavage of C3 (**Figure 1M**). This result agrees with the selectivity of EWE toward fragments of C3. In summary, the observations contradict our prior results with respect to the ability of hC3Nb1 to interact tightly with native C3. Hence, the interaction between hC3Nb1 and native C3 depends on the source of the nanobody and possibly of the purification scheme applied. In particular, it appears that a specific structure at the N-terminus is required since the removal of the N-terminal residue is not sufficient, and the presence of a pyroglutamate rather than a glutamine is incompatible with a high affinity for native C3. Additionally, C3 binding has also been observed by another investigator to vary between variants of hC3Nb1 (personal communication, Alex Macpherson UCB Pharma). We therefore decided to focus further experiments on the EWE variant as its N-terminal extension is likely to make it selective for C3b in a reproducible manner.

### EWE Binding Characteristics

**Figure 2:**
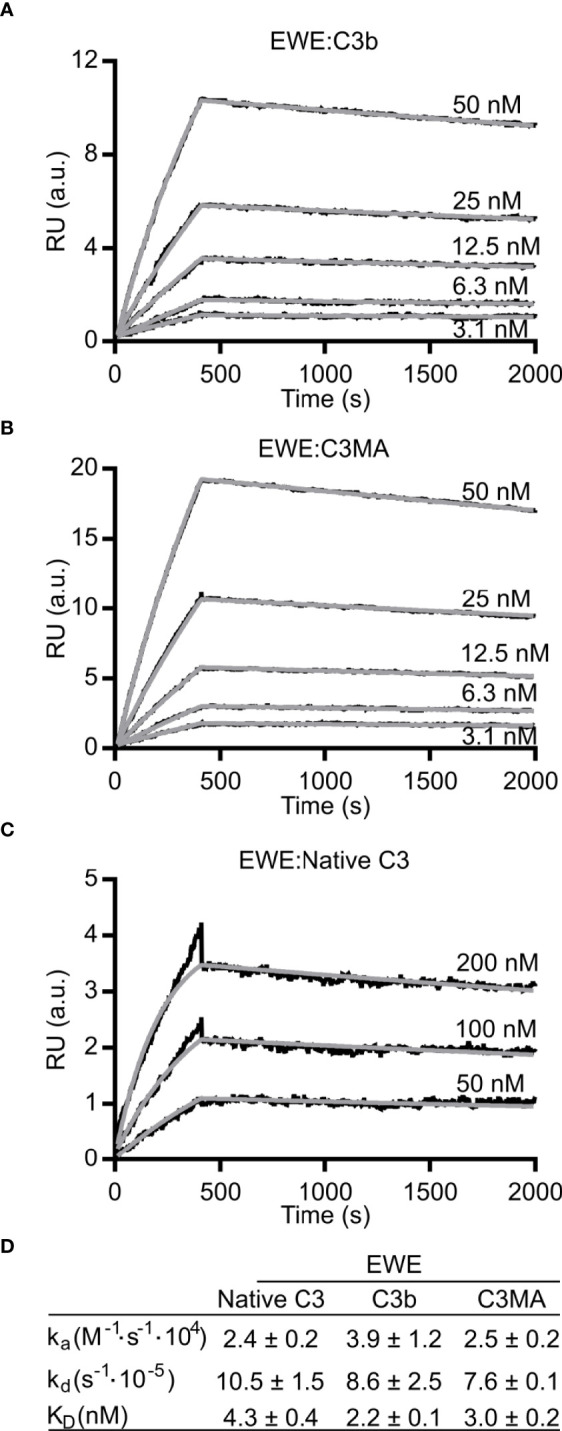
Binding kinetics of EWE. Surface plasmon resonance based analyses of the interaction between EWE and **(A)** C3b, **(B)** C3MA, or **(C)** native C3. **(D)** Table summarizing the binding kinetics of EWE. The averages of rate constants for n=3 experiments are shown. The interaction with native C3 is possibly a result of C3 tick-over during the experiment. *RU*, response units. *a.u.*, arbitrary units. *nM*, nanomolar.

To obtain binding kinetics of the interaction between EWE and the C3 fragments, we immobilized biotinylated EWE on a surface plasmon resonance streptavidin sensor and analyzed the binding of C3b, C3MA, and native C3. When C3b or C3MA were applied to the flow cell, we observed signals with a maximal response of 12-20 RU (**Figures 2A, B**). We fitted the binding curves to a 1:1 binding model and obtained a K_D_ of 2.2 nM and 3.0 nM for C3b and C3MA, respectively. In contrast to the large response of C3MA and C3b, applying native C3 resulted in 3-5 times lower responses despite a four-fold higher concentration of C3 (**Figure 2C**). For native C3, 50 nM was required to give a 1 RU response at 500 s, whereas 50 nM of C3MA elicited a response of 20 RU. We were able to fit these data to a 1:1 binding model and obtain an apparent K_D_ of 4.3 nM; however, we suspect this apparent tight binding to arise from tick-over of C3 occurring during the experiment itself, which was performed at 25°C. **Figure 2D** summarizes the rate constants of association and dissociation of EWE.

### EWE FH Fusion Restores C3b Cleavage

**Figure 3:**
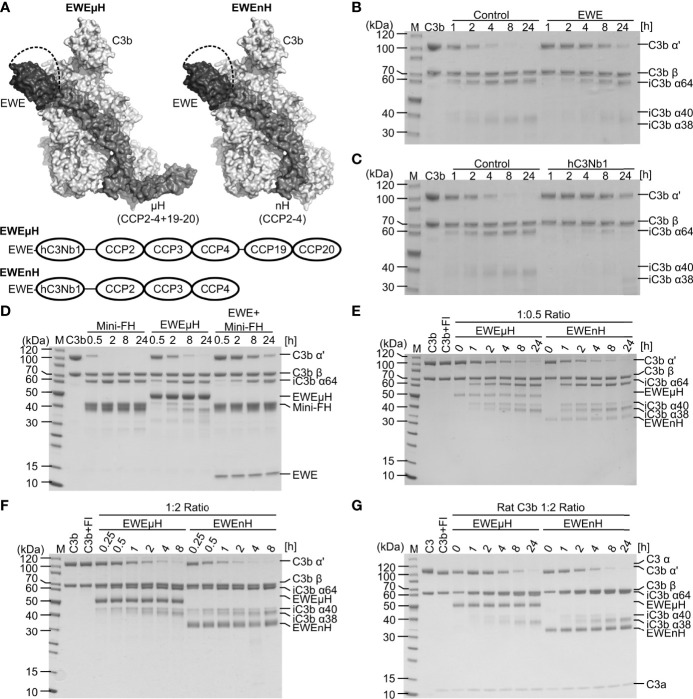
Design and characterization of EWEµH and EWEnH. **(A)** Model of the EWEµH and EWEnH fusion proteins. The crystal structure of hC3Nb1 [PDB entry 6EHG] was superimposed onto the structure of C3b:mini-FH [PDB entry 5O35]. The dashed line illustrates a linker joining the C-terminus of the EWE moiety and CCP2. The lower panel illustrates the domain structure of EWEµH and EWEnH. **(B, C)** Effect of EWE and hC3Nb1 on C3b cleavage. C3b was incubated with FH, FI as well as a 1.2-fold molar excess of either **(B)** EWE or **(C)** hC3Nb1 and the cleavage was monitored by SDS PAGE. **(D)** EWEµH cleavage assay. C3b was incubated with a two-fold molar excess of mini-FH, EWEµH or mini-FH as well as EWE in the presence of FI. The cleavage of C3b was monitored by SDS-PAGE analysis upon the indicated incubation at 37°C. **(E, F)** Comparison of EWEµH and EWEnH. C3b was incubated with FI and EWEµH or EWEnH in molar C3b:EWEµH/EWEnH ratios of **(E)** 1:0.5 and **(F)** 1:2. **(G)** Cleavage assay of rat C3b performed using a molar C3b:EWEµH/EWEnH ratio of 1:2. The C3b was generated from rat C3 using CVFBb and was immediately used for the cleavage assay, explaining the presence of C3a in the reactions. *M*, molecular weight marker.

Previously, we have shown that hC3Nb1 inhibits FH-mediated cleavage of C3b by FI (16). If used in an *in vivo* setting, the inhibition of C3b-degradation by hC3Nb1 may lead to the buildup of C3b on activating surfaces. Hence, we set out to develop an EWE derivative that also allows FI degradation of C3b to make the inhibition irreversible. The engineered, minimized version of FH comprising the CCP domains 1-4 and 19-20, referred to as mini-FH, sustains degradation of C3b by FI (13). A comparison of the crystal structures of the hC3Nb1:C3b (16) and mini-FH:C3b (32) complexes revealed that hC3Nb1 only overlaps with the CCP1 domain of mini-FH. We hypothesized that this binding site would be compatible with the linking of EWE to the CCP2 domain of FH. We designed the fusion protein EWEμH with the C-terminus of EWE linked to FH CCP2-4 and further to CCP19-20, whereas in EWEnH only FH CCP2-4 are included (**Figure 3A**).

We assessed whether EWE inhibits FH-assisted FI cleavage of C3b, similar or different than the parental hC3Nb1. We incubated C3b with FI as well as FH in the presence or absence of EWE or hC3Nb1 and monitored C3b cleavage by SDS-PAGE analyses. In the absence of the nanobodies only trace amounts of C3b remained after 4 h of incubation, whereas a large proportion of the C3b remained non-cleaved after 24 h of incubation in the presence of EWE or hC3Nb1 (**Figures 3B, C**). Hence, as expected both nanobodies inhibit FI-mediated degradation of C3b.

Secondly, we tested the ability of EWEµH to mediate FI cleavage of C3b and compared the effect to mini-FH as well as a mixture of mini-FH and EWE. We mixed C3b with EWEµH and FI and monitored the resulting cleavage of C3b by SDS-PAGE. Only trace amounts of the C3b remained upon 30 min of incubation with mini-FH, whereas incubation with mini-FH in presence of EWE delayed the cleavage (**Figure 3D**). This observation agrees with the steric overlap between EWE and the CCP1 domain of mini-FH. In contrast, the EWEµH fusion protein partially restored C3b cleavage at a C3b:EWEµH ratio of 1:2 (**Figure 3D**).

Finally, we compared EWEnH to EWEµH in a side-by-side cleavage assay, in which we incubated C3b with FI and the two fusion proteins in C3b:fusion protein ratios of 1:0.5 (**Figure 3E**) or 1:2 (**Figure 3F**). These analyses revealed that both fusion proteins sustained the degradation of C3b by FI. Importantly, degradation depended on the fusion proteins since FI did not cleave C3b in the absence of the fusion protein (**Figure 3F**). The EWEnH appeared to be at least two-fold more efficient in serving as a FI cofactor than EWEµH, at both C3b:fusion protein ratios.

To test the cross-reactivity of the two fusion proteins with rodent C3b, we purified C3 from rat plasma and generated C3b through CVFBb mediated cleavage. We mixed rat C3b with fusion protein at a molar ratio of 1:2 and monitored the cleavage at 37°C (**Figure 3G**). Compared to the cleavage of human C3b, the cleavage of rat C3b was slower and revealed the presence of residual C3b α’ chain upon 8 hours of incubation. Similar to the degradation of human C3b, the EWEnH appeared more efficient than EWEµH, as judged by the band intensities of the C3b α’ chain upon 8 hours of incubation. In summary, both fusion proteins possess cofactor activity and sustains FI degradation of both human and rat C3b.

### Affinity of FH Constructs

**Figure 4:**
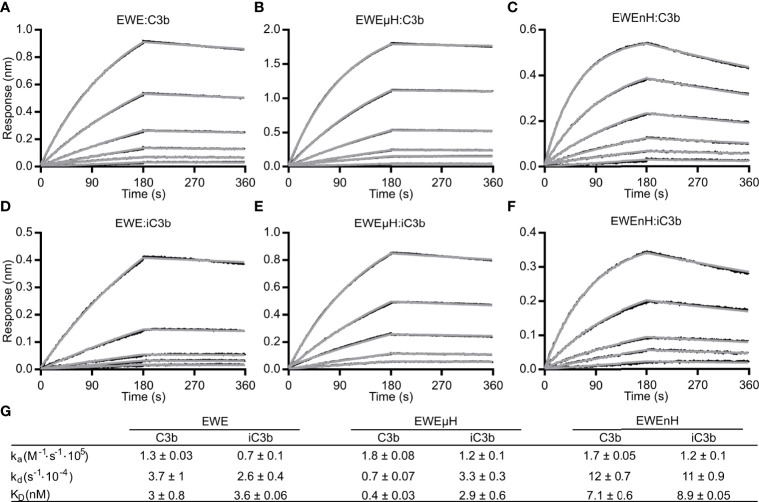
Bio-layer interferometry-based analyses of EWE fusion proteins. **(A)** EWE, **(B)** EWEµH or **(C)** EWEnH was immobilized on a biosensor *via* the C-terminal 6xHis-tag and transferred into dilution series of **(A, B)** 1.5625-50 nM C3b, and **(C)** 3.125-100 nM C3b. Similarly, **(D)** EWE, **(E)** EWEµH or **(F)** EWEnH was immobilized on biosensors and transferred into dilution series of **(D, E)** 3.125-50 nM iC3b or **(F)** 6.25-100 nM iC3b. **(G)** The table summarizes the rate constants of association and dissociation from n=3 experiments for C3b kinetics and n=2 experiments for iC3b kinetics. *nm*, nanometer.

In our initial characterization, we observed a slower C3b degradation in the presence of EWEµH compared to mini-FH (**Figure 3D**). This may arise either from a high affinity of EWEµH towards fragments of C3b, which would limit recycling of the fusion protein, or a steric clash between the EWE moiety and the CCP2 domain, which would delay FI docking onto C3b. We employed BLI and analyzed the affinity of EWE, EWEµH and EWEnH to C3b (**Figures 4A–C**) as well as iC3b (**Figures 4D–F**). We compared the affinity of the fusion proteins to C3b and the iC3b, because high affinity toward the degradation product iC3b potentially limits recycling of the fusion proteins. We compared the fusion proteins to EWE, which exhibited comparable affinities toward C3b and iC3b (K_D_ 3 and 3.6 nM, respectively). This observation agrees with the structural similarity of the EWE epitope between C3b and C3c. EWEµH exhibited a higher affinity toward C3b than EWE (K_D_ 0.4 nM vs 3 nM), indicating that the FH moiety of EWEµH contributes to the interaction with C3b. Furthermore, EWEµH exhibited a 10-fold lower affinity toward iC3b compared to C3b (K_D_ 2.9 vs 0.4 nM). This agrees with the involvement of the CCP19-20 domains, which interacts with the thioester domain of C3b that in iC3b is released from the MG1 domain interaction present in C3b (33, 34). In contrast, EWEnH was largely unaffected by the difference between iC3b and C3b and exhibited comparable affinities towards the two species (K_D_ 7.1 nM vs 8.9 nM). This observation indicates that the primary interaction site of EWEnH lies in the MG-ring region of C3b and hence remains unaffected by FI degradation. Collectively, these data show that EWEµH and EWEnH retain high affinity toward iC3b, which may limit the recycling of the fusion proteins upon the cleavage of C3b to iC3b, but EWEnH dissociates significantly faster from iC3b than both EWE and EWEμH (**Figures 4F, G**). Importantly, both fusion proteins sustained full cleavage when incubated with a molar excess of C3b (**Figure 3E**), indicating that both fusion proteins recycle.

### Functional Comparison of EWE Constructs

**Figure 5:**
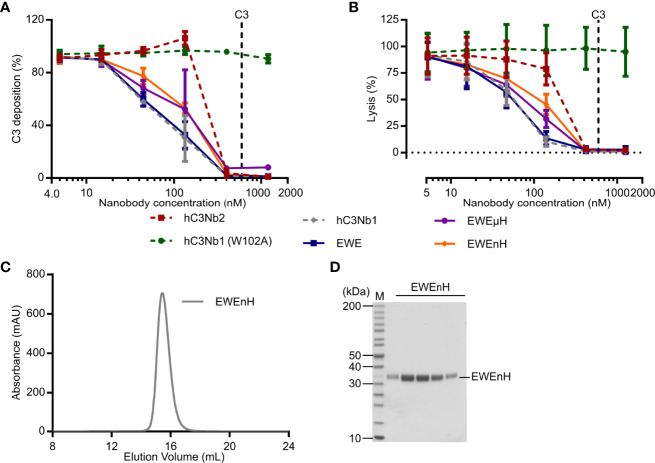
Functional comparison of EWEµH and EWEnH in serum conditions. The effects of EWE, EWEµH, and EWEnH on **(A)** AP mediated deposition of C3 fragments onto a surface of zymosan or **(B)** AP mediated hemolysis of rabbit erythrocytes. In panels A and B, the effects were assayed in 11% human serum and data were normalized to 100% in serum without nanobodies, and 0% without serum. The effects were compared to the parental AP inhibitor hC3Nb1 (16), the broad inhibitor hC3Nb2 (22), and the inactive hC3Nb1 (W102A) mutant (16). Dashed lines indicate putative C3 concentrations assuming a C3 concentration of 5.4 µM in undiluted human serum (17). Average and S.D. (error bars) are shown for n=3 experiments in both panels. **(C)** Ultrafiltration assay. The EWEnH was concentrated to 22 mg/mL and was subjected to SEC. **(D)** SDS-PAGE analysis of peak fractions from panel **(C)** *mAU*, milli absorbance units. *M*, molecular weight marker.

Next, we tested whether EWE, EWEuH, and EWEnH retained their ability to inhibit the AP in serum, using a zymosan based AP activity assay. We compared the effect of the three proteins to the parental hC3Nb1, the inactive hC3Nb1 (W102A) (16), and the broad inhibitor hC3Nb2 (22). We observed that hC3Nb1, EWE, EWEµH, and EWEnH – were equally efficient in inhibiting the AP (**Figure 5A**). These data indicate that fusing FH fragments to EWE does not compromise its inhibitory effect on the AP. Furthermore, all four active hC3Nb1 derivatives inhibited the AP mediated C3 fragment deposition at lower concentrations than hC3Nb2. This agrees with the selective binding of the hC3Nb1 derivatives to C3b, whereas hC3Nb2 binds both native C3 and C3b with equal affinity (22). We also analyzed the effect of EWE, EWEµH and EWEnH on the inhibition of AP mediated lysis of rabbit erythrocytes. This demonstrated that all the hC3Nb1 derivatives inhibited the lysis of rabbit erythrocytes to a similar extent (**Figure 5B**). These data consolidate the fusion proteins as potent inhibitors of the AP and show that the inhibitory function of the proteins have an impact on AP mediated terminal pathway activation.

An important aspect of EWEnH or EWEµH is whether these fusion proteins can be concentrated without aggregating prior to, e.g., *in vivo* evaluation. Whereas EWEnH was readily concentrated to 22 mg/mL by ultrafiltration, the EWEµH started to form visually apparent aggregation at 3.75 mg/mL. We subjected the highly concentrated EWEnH to SEC analysis to evaluate any possible aggregation and found that EWEnH eluted as a monodisperse peak (**Figures 5C, D**). Collectively, these data indicate that EWEnH is superior to EWEµH in cofactor activity and stability at high concentrations and thus the most obvious candidate for *in vivo* studies.

## Discussion

The central involvement of complement component C3 as both the substrate and the scaffold of the AP C3 convertase makes C3 an attractive target for complement inhibitors. However, C3 is present in the 1-1.5 mg/mL range in plasma (17, 18), and efficient complement inhibition through C3 hence requires high doses of C3-targeting therapeutics *in vivo*. The optimal C3-specific inhibitor in the *in vivo* setting thus only bind C3b and allows FI degradation. In the present study, we described the EWE nanobody, which constitutes a rationally engineered version of the previously described hC3Nb1. The development of this nanobody adds to our toolbox of C3 targeting nanobodies, that besides hC3Nb1 (16), comprises the broad complement inhibitor hC3Nb2 (22), and the AP inhibitor hC3Nb3 (35). Our SEC and BLI data indicate that insertion of the ‘EWE’-motif at the N-terminal renders the nanobody incapable of binding C3, whereas it does not affect the binding of C3b. Our functional assessment indicated that the EWE potently inhibits the AP, similarly to the parental hC3Nb1 nanobody. In *in vitro* cleavage assays, we showed that EWE inhibited the cleavage of C3b by FI. We further describe that fusion with fragments of mini-FH, allowed the resulting EWEµH and EWEnH to mediate degradation of C3b.

A surprising observation of our study, was the selectivity of the parental hC3Nb1 toward C3b, contradicting our previous observation that the nanobody binds native C3 with a dissociation constant of 890 pM (16). Based on the spatial proximity of the N-terminus and the MG6 domain in native C3, we suggest that this irreproducible behavior originates from the N-terminal residue of the nanobody, but we have not been able to restore binding to native C3 even by truncation of the nanobody. This suggests that a very specific chemical structure of the N-terminal residue is required for high-affinity binding of native C3. Our data suggests that that subtle differences in the expression and purification protocol of the parental hC3Nb1 nanobody can lead to preparations that either show strong or no interaction with native C3. This underlines the need for thorough characterization of C3 binding prior to the use of hC3Nb1. However, these variations in specificity do not alter the AP inhibitory effect, since the nanobody will inhibit the AP by binding to C3b regardless of whether it interacts with native C3. The interaction with native C3, however, affects the concentration of hC3Nb1 required to obtain full inhibition in plasma and may confer an inhibitory effect on C3 cleavage by the CP/LP C3 convertase. Extending the N-terminus of hC3Nb1 by the EWE-motif prevents the interaction with native C3.

The specificity of EWE resembles the Genentech S77 antibody, which also binds the MG6-MG7 region of C3b (36). Similar to EWE, the binding mode of S77 results in a steric clash between antibody and MG6 in native C3, which renders the S77 antibody specific for fragments of C3, and the S77 antibody also interferes with FH mediated cleavage of C3b by FI (36). In the context of complement therapeutics, nanobodies and antibodies constitute complementary approaches to dampen excessive activation, with different advantages and disadvantages (reviewed in (20)). One major advantage of EWE is its small size, which readily allowed recombinant fusion to CCP domains of FH. Furthermore, the small size may facilitate adeno-associated virus delivery to localized sites, as recently demonstrated for other complement inhibitors (37, 38). The small size of EWEµH and EWEnH likely leads to a short circulation time of the proteins similar to the short circulation time observed for the Newcastle mini-FH (15). Different strategies allow the extension of the circulation time of therapeutic agents. This was demonstrated by Yang et al, who fused the Newcastle mini-FH to FH-related protein domains R1-2 to promote homodimerization. This strategy increased the circulation time more than fivefold and may be adapted to increase the circulation time of EWEnH (39). Alternatively, the circulation time could be increased by fusion to albumin binding nanobodies (40).

The lack of the CCP19-20 of EWEnH compared to the EWEµH construct will reduce the specificity toward self-surfaces and EWEnH will not discriminate self-surfaces from e.g. bacterial surfaces. However, the affinity of EWEµH toward C3b may be sufficient for the protein to bind bacteria opsonized with C3 fragments. This is also true for other components, including compstatin derivatives. The FDA recently approved the C3-specific compstatin derivate Pegcetacoplan (brand name Empaveli) for treatment of PNH (41), which underscores C3 as a feasible therapeutic target. Similarly, the complement component C5 specific antibodies eculizumab and ravulizumab do not distinguish self from intruding pathogens, further substantiating that complement inhibition outweighs the increased risk of infections.

In conclusion, we present the EWE nanobody, which demonstrates rational structure-based engineering of the hC3Nb1 to render the nanobody specific toward C3b and fragments thereof. The nanobody retains its potent AP-inhibitory function and may emerge as a new tool for studies of complement in disease models. We further present an engineered version of EWE, EWEnH, which allows the degradation of C3b and hence pose an important improvement of the nanobody.

## Data Availability Statement

Diffraction data and coordinates for the C3b-EWE complex is available at the protein data bank under https://www.wwpdb.org/pdb?id=pdb_00007qiv.

## Author Contributions

HP: Conceptualization, Investigation, Writing original manuscript. RJ: Supervision, investigation, conceptualization. SP, AH: Investigation. NL: conceptualization, supervision. ST: supervision. GA: conceptualization, supervision, writing original manuscript. All authors contributed to the article and approved the submitted version.

## Funding

GA was supported by the Lundbeck Foundation centre BRAINSTRUC (Grant no R155-2015-2666). HP was supported by the Lundbeck Foundation (Grant no R347-2020-2289).

## Conflict of Interest

Authors HP, RJ, NL, ST and GA are listed as inventors on a patent describing the use of EWE, hC3Nb2 and hC3Nb3. Authors HP, RJ, NL and GA have filed the patent application P6053EP00 for the use of EWEµH and EWEnH.

The remaining authors declare that the research was conducted in the absence of any commercial or financial relationships that could be construed as a potential conflict of interest.

## Publisher’s Note

All claims expressed in this article are solely those of the authors and do not necessarily represent those of their affiliated organizations, or those of the publisher, the editors and the reviewers. Any product that may be evaluated in this article, or claim that may be made by its manufacturer, is not guaranteed or endorsed by the publisher.

## Supplementary Material

Supplementary Table 1 Sequence of EWE, EWEnH and EWEµH. TEV site is highlighted in green. Linkers joining EWE and CCP2 are hightligted in grey. Linker joining CCP4 and CCP19 is highlighted in yellow.

Supplementary Table 2 Data collection and refinement statistics. Statistics for the highest-resolution shell are shown in parentheses. The table was generated with phenix.table_one.

Supplementary Figure 1 Crystal structure of the EWE:C3b. **(A)** Cartoon representation of the overall structure of EWE (orange) in complex with C3b (green). **(B)** Zoom on the N-terminus of EWE displaying the 2mF_o_-DF_c_ map contoured at 1 σ. Labels indicate the positions of glutamine 4 in EWE and the MG6 domain of C3b.

Supplementary Figure 2 Analyses of the parental hC3Nb1. **(A)** Non-reducing SDS-PAGE of peak fractions from panels H-J. Mass spectrometric analyses of **(B)** bacterially expressed hC3Nb1, **(C)** bacterially expressed hC3Nb1 (W102A), **(D)** mammalian expressed hC3Nb1, **(E)** bacterially expressed hC3Nb1 (Q1N), and **(F)** bacterially expressed hC3Nb1 (ΔQ1). BLI based analyses binding to 50 nM native C3 or 50 nM C3b by **(G)** bacterially expressed EWE and hC3Nb1, **(H)** mammalian expressed hC3Nb1 and EWE or **(I)** bacterially expressed hC3Nb1 mutants Q1N and ΔQ1.

## Abbreviations

aHUS, atypical hemolytic uremic syndrome; AP, alternative pathway; BLI, bio-layer chromatography; C3MA, complement component C3 methylamine; CCP, complement control protein; CDR, complementarity-determining region; CP, classical pathway; CVF, cobra venom factor; EWE, hC3Nb1 with a N-terminal Glu-Trp-Glu motif; FB, Factor B; FD, Factor D; FH, Factor H; FI, Factor I; LP, lectin pathway; Mini-FH, minimized version of FH; MG, macroglobulin; Nb, nanobody; Nt-α’, amino-terminal segment of the α’ chain; PDB, Protein Data Bank; RU, response units; SEC, size exclusion chromatography.
