# Supplementary material for: Structure-Guided Engineering of a Complement Component C3-Binding Nanobody Improves Specificity and Adds Cofactor Activity
Source: Front Immunol. 2022 Jul 22;13:872536. doi: 10.3389/fimmu.2022.872536 (PMC9352930; doi:10.3389/fimmu.2022.872536)
Supplement: Supplementary Table 1 — Sequence of EWE, EWEnH and EWEµH. TEV site is highlighted in green. Linkers joining EWE and CCP2 are hightligted in grey. Linker joining CCP4 and CCP19 is highlighted in yellow. [file Table_1.pdf]

Table S1 Sequence of EWE, EWEnH and EWEµH. TEV site is highlighted in green. Linkers joining EWE and CCP2 are hightligted in grey. Linker joining CCP4 and CCP19 is highlighted in yellow.

Name	Sequence
EWE	MEWEQVQLVETGGGLVQAGGSLRLSCAASGSIFSINAMGWFRQAPGKEREFVATINR SGGRTYYADSVKGRFTISRDNGKNMVYLQMHSLKPEDTAIYYCAAGTGWSPQTDNE YNYWGQGTQVTVSSHHHHHH
EWEnH	EWEQVQLVETGGGLVQAGGSLRLSCAASGSIFSINAMGWFRQAPGKEREFVATINRSG GRTYYADSVKGRFTISRDNGKNMVYLQMHSLKPEDTAIYYCAAGTGWSPQTDNEYN YWGQGTQVTVSSGGGGSGGGGSGGGGSGGGGSRPCGHPGDTPFGTFTLTGGNVFEY GVKAVYTCNEGYQLLGEINYRECDTDGWTNDIPICEVVKCLPVTAPENGKIVSSAMEP DREYHFGQAVRFVCNSGYKIEGDEEMHCSDDGFWSKEKPKCVEISCKSPDVINGSPISQ KIIYKENERFQYKCNMGYEYSERGDAVCTESGWRPLPSCEEAHHHHHH
EWEµH	EWEQVQLVETGGGLVQAGGSLRLSCAASGSIFSINAMGWFRQAPGKEREFVATINRSG GRTYYADSVKGRFTISRDNGKNMVYLQMHSLKPEDTAIYYCAAGTGWSPQTDNEYN YWGQGTQVTVSSGGGGSGGGGSGGGGSGGGGSRPCGHPGDTPFGTFTLTGGNVFEY GVKAVYTCNEGYQLLGEINYRECDTDGWTNDIPICEVVKCLPVTAPENGKIVSSAMEP DREYHFGQAVRFVCNSGYKIEGDEEMHCSDDGFWSKEKPKCVEISCKSPDVINGSPISQ KIIYKENERFQYKCNMGYEYSERGDAVCTESGWRPLPSCEEAGGGGGGGGGGGGK CGPPPPIDNGDITSFPLSVYAPASSVEYQCQNLYQLEGNKRITCRNGQWSEPPKCLHPC VISREIMENYNIALRWTAKQKLYSRTGESVEFVCKRGYRLSSRSHTLRTTCWDGKLEY PTCAKRENLYFQGHHHHHH
