# Supplementary material for: Structure-Guided Engineering of a Complement Component C3-Binding Nanobody Improves Specificity and Adds Cofactor Activity
Source: Front Immunol. 2022 Jul 22;13:872536. doi: 10.3389/fimmu.2022.872536 (PMC9352930; doi:10.3389/fimmu.2022.872536)
Supplement: Supplementary Table 2 — Data collection and refinement statistics. Statistics for the highest-resolution shell are shown in parentheses. The table was generated with phenix.table_one. [file Table_2.pdf]

Table S2. Data collection and refinement statistics. Statistics for the highest-resolution shell are shown in parentheses. The table was generated with phenix.table_one.

Data collection

Wavelength	0.9763
Resolution range	48.31-2.8 (2.9-2.8)
Space group	P 21 21 2
Unit cell	255.31 64.88 144.72
Total reflections	816285 (83194)
Unique reflections	60284 (5282)
Multiplicity	13.5 (14.1)
Completeness (%)	98.61 (89.10)
Mean I/sigma(I)	11.74 (0.73)
Wilson B-factor	83.24
R-sym	0.2238 (2.725)
$CC_{1/2}$	0.998 (0.495)

Refinement

Reflections used in refinement	59480 (5272)
Reflections used for R-free	1471 (135)
R-work	0.2359 (0.3593)
R-free	0.2742 (0.4072)
Number of non-hydrogen atoms	13155
RMS(bonds)	0.004
RMS(angles)	0.74
Ramachandran favored (%)	96.62
Ramachandran allowed (%)	3.32
Ramachandran outliers (%)	0.06
Rotamer outliers (%)	2.06
Clashscore	2.21
Average B-factor ($Å^2$)	111.13
