# Supplementary material for: Structure-Guided Engineering of a Complement Component C3-Binding Nanobody Improves Specificity and Adds Cofactor Activity
Source: Front Immunol. 2022 Jul 22;13:872536. doi: 10.3389/fimmu.2022.872536 (PMC9352930; doi:10.3389/fimmu.2022.872536)
Supplement: Supplementary Figure 1 — Crystal structure of the EWE:C3b. (A) Cartoon representation of the overall structure of EWE (orange) in complex with C3b (green). (B) Zoom on the N-terminus of EWE displaying the 2mFo-DFc map contoured at 1 σ. Labels indicate the positions of glutamine 4 in EWE and the MG6 domain of C3b. [file Image_1.pdf]

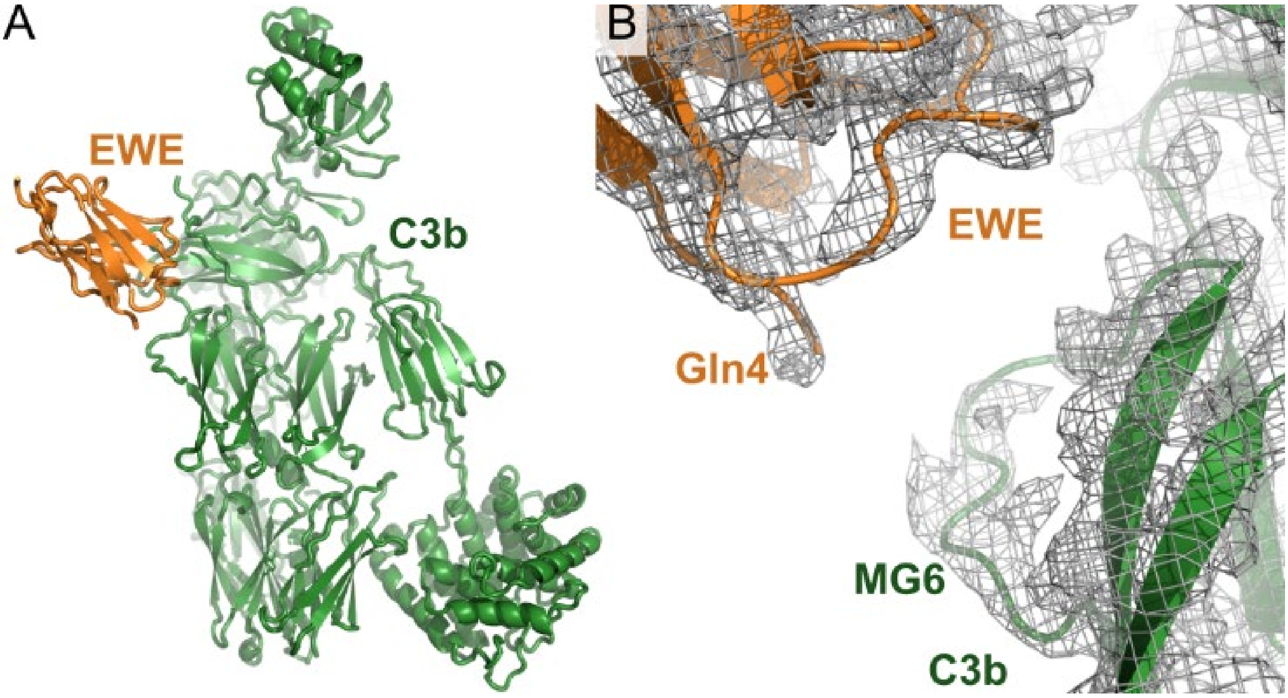

Figure S1 Crystal structure of the EWE:C3b. (A) Cartoon representation of the overall structure of EWE (orange) in complex with C3b (green). (B) Zoom on the N-terminus of EWE displaying the $2mF_o$-DF_c map contoured at 1 σ. Labels indicate the positions of glutamine 4 in EWE and the MG6 domain of C3b.
