# Supplementary material for: Structure-Guided Engineering of a Complement Component C3-Binding Nanobody Improves Specificity and Adds Cofactor Activity
Source: Front Immunol. 2022 Jul 22;13:872536. doi: 10.3389/fimmu.2022.872536 (PMC9352930; doi:10.3389/fimmu.2022.872536)
Supplement: Supplementary Figure 2 — Analyses of the parental hC3Nb1. (A) Non-reducing SDS-PAGE of peak fractions from panels H-J. Mass spectrometric analyses of (B) bacterially expressed hC3Nb1, (C) bacterially expressed hC3Nb1 (W102A), (D) mammalian expressed hC3Nb1, (E) bacterially expressed hC3Nb1 (Q1N), and (F) bacterially expressed hC3Nb1 (ΔQ1). BLI based analyses binding to 50 nM native C3 or 50 nM C3b by (G) bacterially expressed EWE and hC3Nb1, (H) mammalian expressed hC3Nb1 and EWE or (I) bacterially expressed hC3Nb1 mutants Q1N and ΔQ1. [file Image_2.pdf]

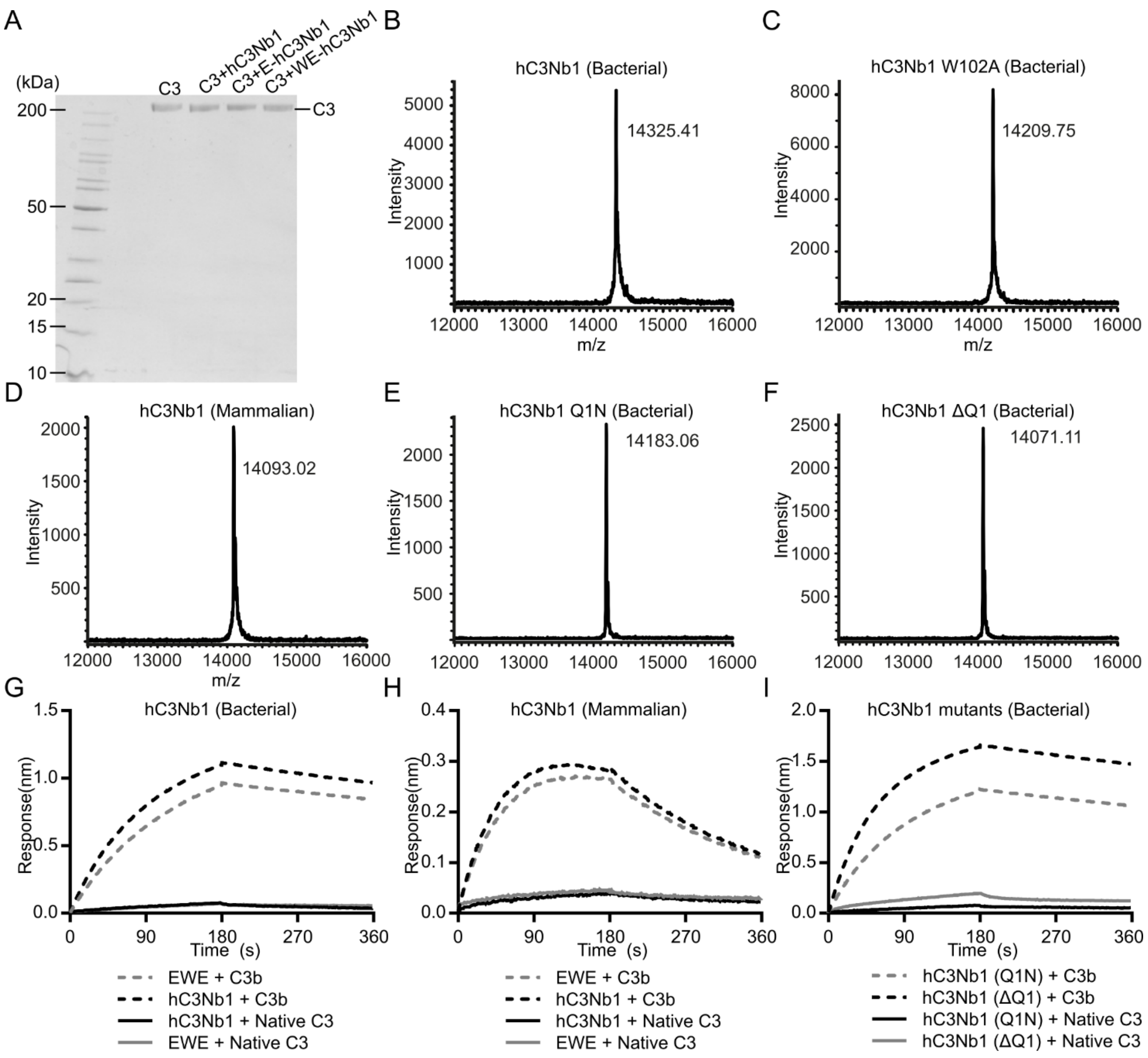

Figure S2 Analyses of the parental hC3Nb1. (A) Non-reducing SDS-PAGE of peak fractions from Figure 1 panels H-J. Mass spectrometric analyses of **(B)** bacterially expressed hC3Nb1, **(C)** bacterially expressed hC3Nb1 (W102A), **(D)** mammalian expressed hC3Nb1, **(E)** bacterially expressed hC3Nb1 (Q1N), and **(F)** bacterially expressed hC3Nb1 (ΔQ1). BLI based analyses binding to 50 nM native C3 or 50 nM C3b by **(G)** bacterially expressed EWE and hC3Nb1, **(H)** mammalian expressed hC3Nb1 and EWE or **(I)** bacterially expressed hC3Nb1 mutants Q1N and ΔQ1.
